# Perceived stress and associated factors among university students in Ethiopia during the late stage of the COVID-19 pandemic: A cross-sectional study

**DOI:** 10.3389/fpsyg.2022.978510

**Published:** 2022-11-03

**Authors:** Wudneh Simegn, Lamrot Yohannes, Abdulwase Mohammed Seid, Asmamaw Emagn Kasahun, Faisel Dula Sema, Adane Flatie, Asrat Elias, Henok Dagne

**Affiliations:** ^1^Department of Social and Administrative Pharmacy, School of Pharmacy, University of Gondar, Gondar, Ethiopia; ^2^Department of Environmental and Occupational Health and Safety, Institute of Public Health, University of Gondar, Gondar, Ethiopia; ^3^Department of Clinical Pharmacy, School of Pharmacy, University of Gondar, Gondar, Ethiopia; ^4^Department of Pharmaceutics, School of Pharmacy, University of Gondar, Gondar, Ethiopia; ^5^Department of Pharmaceutical Chemistry, School of Pharmacy, University of Gondar, Gondar, Ethiopia

**Keywords:** COVID-19, perceived stress, university students, associated factors, Ethiopia

## Abstract

**Background:**

During extensive outbreaks of infectious diseases, people who are impacted, particularly the subgroups of the community who are at an increased risk of mental health problems, may experience increased stress and mental health difficulties. University students are one such susceptible population and are prone to experiencing high levels of stress as compared with the general population. Therefore, this study aimed at assessing perceived stress and identifying its associated factors among university students in Ethiopia during the late stage of the COVID-19 pandemic.

**Methods:**

A cross-sectional study was conducted among university students in Ethiopia from 30 May to 30 June 2021. Students were asked to fill out an online survey on Google Forms that included consent, sociodemographic information, the UCLA-8 Loneliness Scale, the standard validated stress scale (PSS-10) questionnaire, and the three-item Oslo Social Support Scale (OSSS-3) to assess social support. The collected data were exported to SPSS 26. Descriptive and analytical statistics were carried out. Binary and multiple logistic regression analyses were performed to find associated factors, and variables with a *p*-value of 0.05 were considered statistically significant variables.

**Results:**

A total of 426 university students were included in the survey, among whom 268 (62.9%) were male participants. The age of the participants ranged from 18 to 37 years. Health-related departments accounted for 37.1% of the participants, while non-health-related departments accounted for 62.9%. The prevalence of stress was 18.3% in the study population. In this study, extreme susceptibility to COVID-19, sleeping problems, poor self-efficacy to prevent COVID-19, and loneliness were significantly associated with perceived stress.

**Conclusion:**

Stress was prevalent among university students in Ethiopia during the late stage of the COVID-19 pandemic. Extreme susceptibility to COVID-19, sleeping problems, poor self-efficacy, and loneliness were identified as factors for stress. Therefore, we suggest that universities should provide opportunities for safe social connection, counseling, and guidance for students.

## Introduction

Coronavirus disease (COVID-19) is a mild-to-severe respiratory illness transmitted chiefly by contact with infectious materials causing fever, cough, shortness of breath, myalgia or fatigue, pneumonia, dyspnea, headache, diarrhea, hemoptysis, and runny nose (Merriam-Webster, 1984; Staff, 2004). It is a new strain that was first discovered in 2019 in Wuhan, China (Lai et al., 2020; Zhu et al., 2020). COVID-19 was declared a global pandemic by the World Health Organization on 11 March 2020, after infecting over 51,476,042 million persons and killing over 6,266,169 people worldwide (Gilbert et al., 2020a,b; Organization, 2020). In half of January 2021, over 98 million infections were recorded globally, claiming the lives of over 2.1 million people (Winter and Hegde, 2020). The first incidence in Ethiopia was confirmed in Addis Ababa on 31 2020, with 470,587 cases and 7,510 deaths since then (Covid et al., 2020; Gilbert et al., 2020a,b).

Psychological symptoms such as anxiety/stress, panic buying, fear and paranoia about attending community events, reduced autonomy and concerns about income, job, and security have been observed (Zhou et al., 2020). Because of the extensive disruption caused by COVID-19, numerous countries have implemented various prevention and control measures, including quarantining, closing, avoiding public meetings, and even holding various public service activities, such as education (Barua, 2020; Khan and Faisal, 2020). Over 1.5 billion school children and young people were compelled to stay at home, causing great fear, uncertainty, and stress (Coalition, 2020; Husain, 2020). University students are especially prone to feelings of loneliness and experiencing higher rates of anxiety/stress and depression than the general population (Rahman et al., 2012; Diehl et al., 2018).

Worldwide, many studies have assessed the level of perceived stress among university students before the pandemic (Shah et al., 2010; Borjalilu et al., 2015; Gazzaz et al., 2018; Al-Qahtani and Alsubaie, 2020; Karyotaki et al., 2020; Tariq et al., 2020). However, a limited number of studies on the same topic have been conducted during and after the COVID-19 outbreak. The available studies reported a high perceived stress level among students, with levels ranging from 12.6 to 30.2% (AlAteeq et al., 2020; Pedrozo-Pupo et al., 2020; Sheroun et al., 2020). For example, the perceived stress level was recorded to be about 25% in China (Cao et al., 2020). In Spain, about 41% of students had stress symptoms during the early phase of the COVID-19 pandemic (Rodríguez-Rey et al., 2020), and another study involving university students reported about 32.5% of COVID-19-related stress (Aylie et al., 2020). In Ethiopia, the prevalence of stress among university students was reported to be 28.6% (Simegn et al., 2021), and the perceived stress among healthcare providers was reported to be 51.6% in the early stage COVID-19 (Chekole et al., 2020).

As previously suggested (Chang et al., 2020), identifying the risk and other associated factors is crucial for the development of new guidelines and focused interventions to help students. It is highly critical to comprehend and investigate the psychological situations of university students during this chaotic period. The hypothesis of this research is that the COVID-19 pandemic and other related factors increase the risk of high perceived stress level of university students. This study may help create a better approach to promote mental health and wellbeing among health professional students in the late stage of the COVID-19 pandemic. The findings of this study will inspire stakeholders to act by delivering baseline information on the prevalence of perceived stress and identifying associated causes. Therefore, this study aimed at determining the level of perceived stress and examining the role of sociodemographic and COVID-19-related characteristics as potential determinants of stress in a sample of Ethiopian undergraduate students during the late stage of the COVID-19 pandemic.

## Methods

### Study design, setting, and period

This cross-sectional study was conducted among university students from 30 May to 30 June 2021 in Ethiopia.

### Study population and eligibility criteria

**Figure 1:**
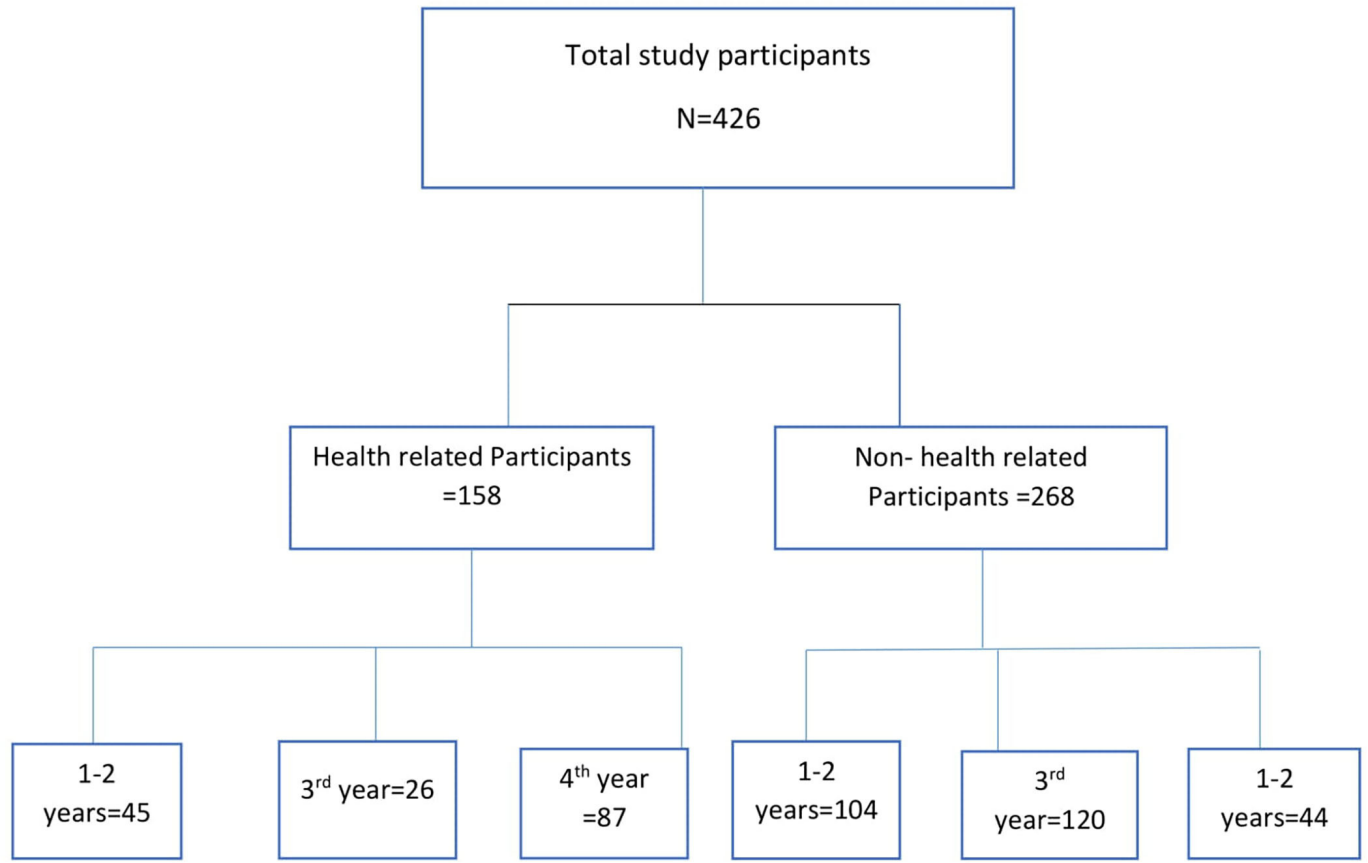
Schematic presentation of study participants.

All university students who had used social media (e.g., Telegram, Facebook, and Imo); volunteered, granted ethical consent, and were available online during the study period were included (Figure 1). The survey was voluntary-based, which included the participant consent form attached with online instruments at the beginning of the questionnaire.

### Sampling technique and sample size determination

All study participants who can access online Google Forms during the study period were included by using the snowball sampling method. A total of 426 study participants filled out the questionnaire within 1 month.

### Data quality control

The questionnaires were checked for consistency, completeness, clarity, and accuracy. A pretest was conducted among 20 residents who were later excluded from the study. Minor modifications were amended based on the findings of the pretest.

### Data collection tool and procedure

The data collection tool used in the present study consists of three parts: The first part consists of questions on sociodemographic characteristics, the second part consists of questions using loneliness (UCLA-8) scales, and the third part consists of the Perceived Stress Scale (PSS-10) (S1).

The questionnaire was distributed on the social media to university students *via* Telegram groups, Imo, Emails through student representatives, and Facebook. The students were asked to continue the survey once they had read the introduction of the questionnaire, including the purpose of the study, consent to participate, and the confidentiality issue as well as their right to discontinue even if they had started to fill it out.

### Measurement of variables

#### Sociodemographic survey

Sociodemographic variables included in this study were sex (female/male), age in years (18–22/23–37), department (health-related/others), family residence (urban/rural), years of study (1–2 years/3rd year/4+ years), love engagement (yes/no), living alone (yes/no), sexual harassment (yes/no), smoking (yes/no), khat chewing (yes/no), alcohol drink (yes/no), chronic disease (yes/no), daily talk about COVID-19 (yes/no), and daily check of COVID report (yes/no). The questions included were used in previous studies conducted in Ethiopia (AlAteeq et al., 2020; Chekole et al., 2020; Awoke et al., 2021; Charles et al., 2021; Simegn et al., 2021).

#### Sexual harassment

Respondents were asked if they have ever encountered any form of sexual harassment in their lifetime. Those who encountered at least one form of sexual harassment were considered sexual harassed.

#### Smoking, khat chewing, and alcohol use status

Respondents were asked if they have ever smoked cigarettes, chewed chat, and consumed alcohol in their lifetime.

#### Daily talk about COVID-19

Respondents were asked if they have daily talked about COVID-19 with their friends in the preceding month of the data collection with a yes or no option.

#### Check daily report for COVID-19

Respondents were asked if they have checked daily reports for COVID-19 in the preceding month of the data collection with a yes or no option.

#### Sleeping problem

It was assessed by using a single self-report item with a yes or no response. Respondents were asked if they have faced difficulty falling asleep at least once over the week preceding the survey (Do you encounter falling asleep at least once over the previous week?) (Xue et al., 2020). Respondents who answered ‘yes’ were considered having sleeping problems.

#### Extreme susceptibility to COVID-19

It was assessed by using a single self-reported item with a yes or no option (Do you think you are susceptible to COVID-19?). The study participants who answered ‘yes' were considered “extreme susceptible to COVID-19” (Xue et al., 2020).

#### Self-efficacy

To assess the levels of their self-efficacy related to COVID-19, study participants were asked by using a single item with a yes or no answer (Are you confident that you can prevent yourself from getting COVID-19 in case of an outbreak?) (De Zwart et al., 2009). Study participants who answered ‘yes’ were considered to have self-efficacy.

#### Loneliness

The UCLA-8 is used to measure loneliness (McQuaid et al., 2021; Panayiotou et al., 2021). It consisted of the following items: I lack companionship; there is no one I can turn to; I am an outgoing person; I feel left out; I feel Isolation from others; I can find companionship when I want it; I am unhappy being so withdrawn; and People are around me but not with me. These questions were provided with answers “never, rarely, sometimes, and always”. The overall loneliness was scored as follows: 1 for never, 2 for rarely, 3 for sometimes, and 4 for always. The total score obtained by the participants was between 8 and 32. The scores of the eight-item scale were categorized by degrees of loneliness: none (8–16), mild (17–20), moderate (21–24), or severe (> 24) (Hays and DiMatteo, 1987; Greene et al., 2018). Finally, study participants who reported mild, moderate, and severe loneliness were categorized as having loneliness. The Cronbach alpha for loneliness scales in the current study was 0.812.

#### Social support

The three-item Oslo Social Support Scale (OSSS-3) was used to assess the level of social support received (Bøen et al., 2012; Kocalevent et al., 2018). The three items assessed the number of close confidants (1. How many people are so close to you that you can count on them if you have great personal problems? (Write the number of people as 0, 1, 2...); perceived level of concern from others (2. How much interest and concern do people show in what you do?); and perceived ease of getting help from neighbors (3. How easy is it to get practical help from neighbors if you should need it?). For the first item, study participants were asked to provide the numbers of individuals close to them. The first item was scored based on the number of confidants: 1 for none, 2 for 1, 2, 4 for 3-5, and 4 for 5+ person. The second item was scored as follows: 1 for none, 2 for little, 3 for uncertain, 4 for some, and 5 for a lot. For the third item, study participants were scored as follows: 1 for very difficult, 2 for difficult, 3 for possible, 4 for easy, and 5 for very easy. The total score was 3–14. Scores between 3 and 8 were considered poor, scores from 9 to 11 as moderate, and those from 12 to 14 indicate strong social support (Abiola et al., 2013). The tool was adapted from a previous study in Ethiopia (Asnakew et al., 2021). The Cronbach alpha for the three-item Oslo scale in the current study was 0.732.

#### Perceived stress

The PSS-10 was used to measure the perceived stress level of students. The tool previously validated in a study conducted in Ethiopia showed cultural adaptability of the PSS-10 among Ethiopian university students (Manzar et al., 2019; Awoke et al., 2021). The perceived stress level (In the last month, how often have you been upset because of something that happened unexpectedly?; In the last month, how often have you felt that you were unable to control the important things in your life?; In the last month, how often have you felt nervous and “stressed”?; In the last month, how often have you felt confident about your ability to handle your personal problems?; In the last month, how often have you felt that things were going your way?; In the last month, how often have you found that you could not cope with all the things that you had to do?; In the last month, how often have you been able to control irritations in your life?; In the last month, how often have you felt that you were on top of things?; In the last month, how often have you been angered because of things that were outside of your control?; and in the last month, how often have you felt difficulties were piling up so high that you could not overcome them?). was scored as follows: 0 for never, 1 for almost never, 2 for sometimes, 3 for fairly often, and 4 for very often. The total score was between 0 and 40. A cutoff point of ≥ 20 was considered encountering stress. The Cronbach alpha value for the Perceived Stress Scale in the current study was 0.765, but the authors could not find previous reports on perceived stress.

### Statistical analysis

The data were collected through online Google Forms and exported to SPSS version 26 for analysis. Mean, frequency, and percentage were computed. Logistic regression analysis was used to identify factors associated with perceived stress. As the outcome variable in the current study was categorical (binary outcomes), that is, dichotomized into having symptoms of perceived stress or not having stress and fulfilling other assumptions of logistic regression, it is possible to use logistic regression to identify factors. In this study, perceived stress was the dependent variable, while sex, age, department, family residence, years of study, love engagement, living alone, sexual harassment, smoking, khat chewing, alcohol drinking, chronic disease, talking about COVID-19 daily, checking COVID-19 report daily, sleeping problem, extreme susceptibility to COVID-19, self-efficacy, social support, and loneliness were the independent variables. Binary logistic regression analysis was employed to identify candidate variables for multiple logistic regression. Variables with a *p*-value <0.2 were considered candidate variables: sex, age in years, residence, department, living alone, khat chewing, sleeping problem, extremely susceptible to COVID-19, self-efficacy, and loneliness. These candidate variables were used in the final model and multivariable logistic regression analyses. As there were more than one independent variable, multiple logistic regression was employed. The independent variables having *p*-values <0.05 were judged factors for perceived stress.

## Result

### Sociodemographic characteristics

In this study, 426 university students participated, of which 268 (62.9%) were male students. The mean age was 23.5 (±3.42) years, ranging from 18 to 37 years. About 37.1% of the participants were from health-related departments, and the rest of the study participants were from other non-health-related departments. Most of the study participants (64.1%) come from a family of urban residences (Table 1).

**Table 1:** Socio-demographic characteristics of study participants among university students in Ethiopia, 2021 (*n* = 426).

**Variable**	**Categories**	**Frequency**	**Percent**
Sex	Female	158	37.1
	Male	268	62.9
Age	18–22	179	42.0
	23–37	247	58.0
Department	Health related	158	37.1
	Others	268	62.9
Family residence	Rural	153	35.9
	Urban	273	64.1
Years of study	1–2 years	149	35.0
	3rd year	146	34.3
	4th + years	131	30.8
Love engagement	No	301	70.7
	Yes	125	29.3
Living alone	Yes	134	31.5
	No	292	68.5
Sexual harasmess	No	316	74.2
	Yes	110	25.8
Smoking	No	383	89.9
	Yes	43	10.1
Chat chewing	No	291	68.3
	Yes	135	31.7
Alcohol drink	No	231	54.2
	Yes	195	45.8
Chronic disease	No	374	87.8
	Yes	52	12.2
Do you daily talk about COVID-19	No	285	66.9
	Yes	141	33.1
Do you check COVID-19 is report daily	No	304	71.4
	Yes	122	28.6

### Perceived stress and other conditions

About 263 (61.7%) students had encountered sleeping problems during COVID-19, and about 16.7% of the participants had reported to be extremely susceptible to COVID-19. About 326 (76.5%) students reported loneliness, and 277 students (65.5%) had poor social support. In the current study, 78 (18.3%, 95% CI: 14.8–22.5) university students had symptoms of perceived stress (Table 2).

**Table 2:** Perceived stress and other conditions during late stage of COVID-19 among university students in Ethiopia, 2021 (*n* = 426).

**Variable**	**Categories**	**Frequency**	**Percent**
Sleeping problem	No	263	61.7
	Yes	163	38.3
Extreme susceptibility to COVID-19	No	355	83.3
	Yes	71	16.7
Self-efficacy	No	287	67.4
	Yes	139	32.6
Social support	Poor	277	65.0
	Moderate	124	29.1
	Strong	25	5.9
Loneliness	Yes	326	76.5
	No	100	23.5
Perceived stress	No	348	81.7
	Yes	78	18.3

### Identified factors to perceived stress during the late stage of COVID-19

In the current study, sex, age in years, residence, department, living alone, khat chewing, sleeping problem, extremely susceptible to COVID-19, self-efficacy, and loneliness were candidate variables for the final model and used for multivariable logistic regression analysis. In the final model, extremely susceptible to COVID-19 (AOR = 2.02, 95% CI: 1.02, 4.00), having sleeping problems (AOR = 2.62, 95% CI: 1.43, 4.80), no self-efficacy (AOR = 2.00, 95% CI: 1.04, 3.89), and loneliness (AOR = 4.82, 95% CI: 1.43, 16.22) were significantly associated with perceived stress (Table 3).

**Table 3:** Associated factors of perceived stress, among university students in Ethiopia, 2021 (*n* = 426).

**Variables**	**Categories**	**perceived stress**		**COR (95% UI)**	**AOR (95% UI)**
		**Yes (%)**	**No (%)**		
Sex	Male	42 (15.7)	226 (84.3)	1	1
	Female	36 (22.8)	122 (77.2)	1.59 (0.97, 2.61)	1.47 (0.83, 2.60)
Age in years	18–22	42 (23.5)	137 (76.5)	2.80 (1.10, 2.95)	1.77 (0.83, 2.60)
	23–37	36 (14.6)	211 (85.4)	1	1
Residence	Urban	41 (15.0)	232 (85.0)	1	1
	Rural	37 (24.2)	116 (75.8)	1.81 (1.10, 2.97)	1.50 (0.86, 2.63)
Department	Health related	20 (12.7)	138 (87.3)	1	1
	Others	58 (21.6)	210 (78.4)	1.91 (1.10, 3.31)	1.26 (0.66, 2.41)
Living alone	No	45 (15.4)	247 (84.6)	1	1
	Yes	33 (24.6)	101 (75.4)	1.79 (1.10, 2.97)	1.61 (0.91, 2.86)
Extremely susceptible to COVID-19	No	52 (14.6)	303 (85.4)	1	1
	Yes	26 (36.6)	45 (63.4)	3.37 (1.91, 5.93)	2.02 (1.02, 4.00)^*^
Sleeping problem	No	25 (9.5)	238 (90.5)	1	1
	Yes	53 (32.5)	110 (67.5)	4.59 (2.71, 7.77)	2.62 (1.43, 4.80)^**^
Khat chewing	Yes	33 (24.4)	102 (75.6)	1.77 (1.01, 2.93)	1.25 (0.70, 2.23)
	No	45 (15.5)	246 (84.5)	1	1
Self-efficacy	No	63 (22.0)	224 (78.0)	2.32 (1.27, 4.25)	2.00 (1.04, 3.89)^*^
	Yes	15 (10.8)	124 (89.2)	1	1
Loneliness	Yes	75 (23.0)	251 (77.0)	9.66 (2.98, 31.36)	4.82 (1.43, 16.22)^*^
	No	3 (3.0)	97 (97.0)	1	1

^*^p < 0.05 and ^**^p < 0.01.

## Discussion

The present study assessed the perceived stress and its associated factors among university students during the late stage of the COVID-19 pandemic. In this study, a considerable magnitude (18.3%, 95% CI: 14.8–22.5) of the study participants were identified to have perceived stress. It was lower than that in the study conducted at the early stage of COVID-19 in Ethiopia (Simegn et al., 2021). The difference might be due to the fact that the current study has been conducted at the late stage of COVID-19, and in this study, the PSS-10 was used to measure perceived stress, while the previous study had used the standard validated Depression, Anxiety, and Stress Scale (DASS-21) questionnaire. Perceived stress in this study was also lower than that found in several studies conducted in France (Bourion-Bédès et al., 2021), Turkey (Aslan et al., 2020), the KSA, China (Cao et al., 2020), Iran (Ganjoo et al., 2021), and India (Wakode et al., 2020). Perceived stress in the current study was higher than that identified in a study conducted among Switzerland students (Elmer et al., 2020). The disparity may be due to difference in methods used and different measurement periods/timing in conducting the study. The high level of perceived stress and other mental issues due to COVID-19 can result in difficulties in focusing on academic work (Kecojevic et al., 2020).

Consequently, university students who reported loneliness were about 4.8 times more likely to experience stress, which is consistent with prior research result (Palgi et al., 2020; Ye et al., 2020). This is expected that being separated and distant from their friends and family increases students' risk of stress (Miller et al., 2018). Loneliness during the COVID-19 pandemic may also be largely attributed to or exacerbated by social distancing measures and associated reductions in personal connection with others, leading to increased perceived stress (DiGiovanni et al., 2004; Hoffart et al., 2020).

Because of being lonely for a long period of time without any guidance and support, university students who reported extremely susceptible to COVID-19 were two times more likely to experience stress than their counterparts. This is supported by previous studies: believing extremely susceptible to COVID-19 had an impact on inducing stress problems (Prikhidko et al., 2020), and adopting positive coping strategies can weaken the stress of individuals (Yan et al., 2021).

In addition, students with sleeping problems were 2.5 times more likely to have perceived stress. This is supported by previous studies (Talib and Zia-ur-Rehman, 2012; Doolin et al., 2018; Du et al., 2020; Barutcu Atas et al., 2021; Kara, 2021; Liu et al., 2021; Luo et al., 2021; Zhai et al., 2021; Zhao et al., 2021). As Altena et al. showed sleep quality was also affected by the impact of the COVID-19 pandemic (Altena et al., 2020). Poor-quality or insufficient sleep can lead to maladaptive changes to the stress response (Kara, 2021; Luo et al., 2021). On a biological level, poor sleep quality and sleep deprivation are thought to influence stress-related parameters including cortisol levels and systemic inflammation (Irwin et al., 2016).

The current study showed that students with no self-efficacy were two times more likely to have stress than those with better self-efficacy. This finding is supported by previous research works during COVID-19 (Kara, 2021; Luo et al., 2021; Zhao et al., 2021; Meyer et al., 2022). The impacts of COVID-19 on self-efficacy were also described in several previous studies (Alemany-Arrebola et al., 2020; Pragholapati, 2020; Talsma et al., 2021), including stress among university students (Husky et al., 2020; Lee et al., 2021; Prowse et al., 2021; Hamaideh et al., 2022).

There are few limitations to be noted: The snowball sampling method used in this study does not allow generalizing the results to the totality of Ethiopian university students or the research university. However, since the sample includes students from different campuses (in different municipalities) and from various undergraduate courses at the university, it can be considered broad and representative. A cross-sectional study could have contributed to some bias in the study results. First, selection bias may exist because only students who were familiar with web-based surveys would have responded. Second, self-reported data may be subject to social desirability bias and result in underreporting data. It does not allow causality inference and evaluating the long-term impacts of the pandemic crisis. In the current study, sleeping problems, extreme susceptibility to COVID-19, and self-efficacy were measured by a single question. However, despite these constraints, the results present relevant evidence that can foster future longitudinal studies, and the findings provide baseline data on the prevalence of perceived stress and identified associated factors, motivating stakeholders to take action.

## Conclusion

The results provide insights into how the COVID-19 pandemic and other associated factors affected the mental health and wellbeing of university students. A considerable number of study participants were identified to have perceived stress associated with COVID-19, affecting their mental health and wellbeing. The results demonstrate that the predominance of stress is higher in students, and also more stress was perceived by the students who reported loneliness, have sleeping problems, and have poor self-efficacy. The influence of COVID-19 on students' wellbeing and their education will impact at an extended level, which necessitates an appropriate plan for preparedness. The results of this study are consistent with the hypothesis and highlight the factors that should be taken into account by stakeholders to counteract students' stress and also highlight the need to focus on students during a pandemic.

## Data availability statement

The raw data supporting the conclusions of this article will be made available by the authors, without undue reservation.

## Ethics statement

The studies involving human participants were reviewed and approved by University of Gondar Ethical Review Committee. The patients/participants provided their written informed consent to participate in this study. Written informed consent was obtained from the individual(s) for the publication of any potentially identifiable images or data included in this article.

## Author contributions

All authors made a significant contribution to the work reported, including conception, study design, execution, acquisition, analysis, and interpretation of data. Took part in drafting, revising or critically reviewing the manuscript, gave final approval of the version to be published, have agreed on the journal to which the article has been submitted, and agree to be accountable for all aspects of the work.

## Conflict of interest

The authors declare that the research was conducted in the absence of any commercial or financial relationships that could be construed as a potential conflict of interest.

## Publisher's note

All claims expressed in this article are solely those of the authors and do not necessarily represent those of their affiliated organizations, or those of the publisher, the editors and the reviewers. Any product that may be evaluated in this article, or claim that may be made by its manufacturer, is not guaranteed or endorsed by the publisher.
